# Five ways seascape ecology can help to achieve marine restoration goals

**DOI:** 10.1007/s10980-025-02099-9

**Published:** 2025-06-04

**Authors:** L. M. Wedding, C. E. Stuart, L. L. Govers, R. J. Lilley, A. Olds, J. Preston, L. E. Tavasi, S. J. Pittman

**Affiliations:** 1 School of Geography and the Environment, University of Oxford, Oxford, OX1 3QY UK; 2 Conservation Ecology Group, Groningen Institute for Evolutionary Life Sciences, University of Groningen, PO Box 11103, 9700 CC Groningen, The Netherlands; 3 Department of Coastal Studies, Royal Netherlands Institute for Sea Research, ‘t Horntje, Texel, The Netherlands; 4 School of Science, Technology and Engineering, University of the Sunshine Coast, Maroochydore DC, QLD 4558 Australia; 5 Institute of Marine Sciences, School of the Environment and Life Sciences, University of Portsmouth, Portsmouth, PO4 9LY UK

**Keywords:** Seascape ecology, Marine restoration, Seascape restoration, Connectivity, Cross-habitat facilitation

## Abstract

**Context:**

Marine restoration is increasingly recognized as a key activity to regenerate ecosystem integrity, safeguard biodiversity, and enable ocean sustainability. Global policies such as the Kunming-Montreal Global Biodiversity Framework include area-based targets to improve ecosystem integrity and connectivity. Achieving these targets requires scaling up restoration in ecologically and socially meaningful ways.

**Objectives:**

The objective was to establish a consistent language and framework for seascape restoration practitioners that complements existing marine restoration guidelines and can help to achieve cross-scale restoration targets.

**Methods:**

We proposed that the integration of the 5Cs of seascape ecology—Context, Configuration, Connectivity, Consideration of scale, and Culture— can offer a valuable framework for advancing marine restoration practice and policy. We synthesized existing ecological and social science evidence to demonstrate how the 5Cs framework can be applied to seascape restoration efforts.

**Results:**

We established a consistent language and framework for marine restoration practitioners and recommended four key operational pathways: (1) focusing on the recovery of interconnected habitats across the land–sea interface; (2) integrating the 5Cs from site selection through to monitoring; (3) representing social, historical, cultural, and ecological variables when assessing site suitability; and (4) fostering transdisciplinary collaborations to support integrative, multifaceted projects.

**Conclusions:**

Integrating landscape ecology concepts and methods into coastal restoration will enable the effective scaling up of regenerative actions. Applying the 5Cs can help achieve global restoration targets through more strategic, inclusive, and effective marine restoration across coastal seascapes.

## Introduction

Landscape ecology primarily focuses on the patterns and processes of terrestrial ecosystem structure, function, and change. However, the application of landscape ecology theory and practice in the coastal zone has advanced our ecological understanding at the land-sea interface. For instance, the German biogeographer Carl Troll, who coined the term landscape ecology (*Landschaftsökologie*), mapped and studied the spatial patterns of a mangrove forest in his landmark paper (Troll 1939). Pioneering field experiments tested the Theory of Island Biogeography on mangrove islands (Simberloff and Wilson 1969), and detailed studies of intertidal benthic communities advanced our understanding of coastal patch dynamics (Paine and Levin 1981). These early coastal landscape ecology studies highlighted ecological findings that had implications for understanding coastal ecosystem recovery across a mosaic of habitats.

Seascape ecology, the study of marine spatial and temporal patterns and their ecological consequences, later emerged as a new scientific conceptual framework for shallow coastal seascapes (Ray 1991; Jones and Andrew 1992; Robbins and Bell 1994) and broader-scale oceanic seascapes (Steele 1989). The discipline of seascape ecology has since evolved from a fusion of concepts and tools from landscape ecology, quantitative marine ecology, and the geosciences (Boström et al. 2011; Wedding et al. 2011). The term *seascape* has been defined from a systems perspective as a spatially heterogeneous and dynamic marine space that can be delineated at a wide range of scales in time and space (Pittman 2018), where human influence is integral to a system but not necessarily central.

Restoration efforts are increasingly recognized as essential to addressing the global decline of coastal ecosystems (Duarte et al. 2020). Well-designed restoration can enhance biodiversity, recover degraded habitats, support nature’s contributions to people, build environmental stewardship, and improve human well-being (Saunders et al. 2020; Sievers et al. 2022; Manea et al. 2023). Global policies, such as the Convention on Biological Diversity’s Kunming-Montreal Global Biodiversity Framework (GBF) Target 2 (CBD 2022), set area-based restoration targets prioritizing ecological integrity and connectivity. Regionally, the European Union’s (EU) Nature Restoration Law mandates restoring 20% of the EU's degraded terrestrial, freshwater, and marine ecosystems by 2030 (European Commission 2024). The law emphasizes restoring connectivity through interventions that enhance natural flows, especially in surface waters and wetlands, to support species movement, climate adaptation, and population viability. Furthermore, the EU Nature Restoration Law specifically links enhancements to connectivity and ecological coherence with improving the quality and quantity of habitat for listed marine species and wild birds. Globally, ecological connectivity is included as a key indicator in GBF Target 2, highlighting its role in maintaining habitats and achieving long-term restoration goals. As such, these policy targets are more closely aligned than ever with landscape ecology principles and practices. Consequently, there is now a pressing need for science to support the scaling up of restoration efforts in ecologically, socially, and historically meaningful ways to regenerate ecological functions for a healthy and resilient biosphere for all species (Suding et al. 2015; Gilby et al. 2018; Cohen-Shacham et al. 2019; McAfee et al. 2022).

Seascape restoration is an emerging holistic approach for restoring degraded marine ecosystems that explicitly considers the intrinsic connectivity and interdependence among the mosaic of habitats we refer to as the seascape ﻿(Preston et al. 2025). In addition, seascape restoration should consider the social-ecological system, including the related connectivity and interdependence among different species (including humans), habitats, and ecosystems.This approach emphasizes the recovery of habitat functionality and ecosystem resilience that is fundamentally underpinned by dynamic interactions between marine and terrestrial, social and ecological processes. The science of seascape restoration is grounded in ecological theory, with significant influences from landscape ecology, restoration ecology, and ecological engineering (Bell et al. 1997). Seascape restoration encompasses both passive and active restoration while recognizing that in many situations, passive conservation (safeguarding and unassisted recovery) approaches alone may not be sufficient to regenerate ecosystem integrity in the face of ecosystem modification, loss, and dysfunction (Olds et al. 2016). The most significant advances in applying seascape ecology to coastal restoration have been made through decades of experience with wetland restoration. For example, several studies have prioritized key spatially explicit processes as important context-dependent considerations for restoration site allocation, such as the filtration of land-based nutrients by fringing mangroves at the land-sea interface (Zedler 2000; Simenstad et al. 2006; Weinstein 2007).

**Fig. 1:**
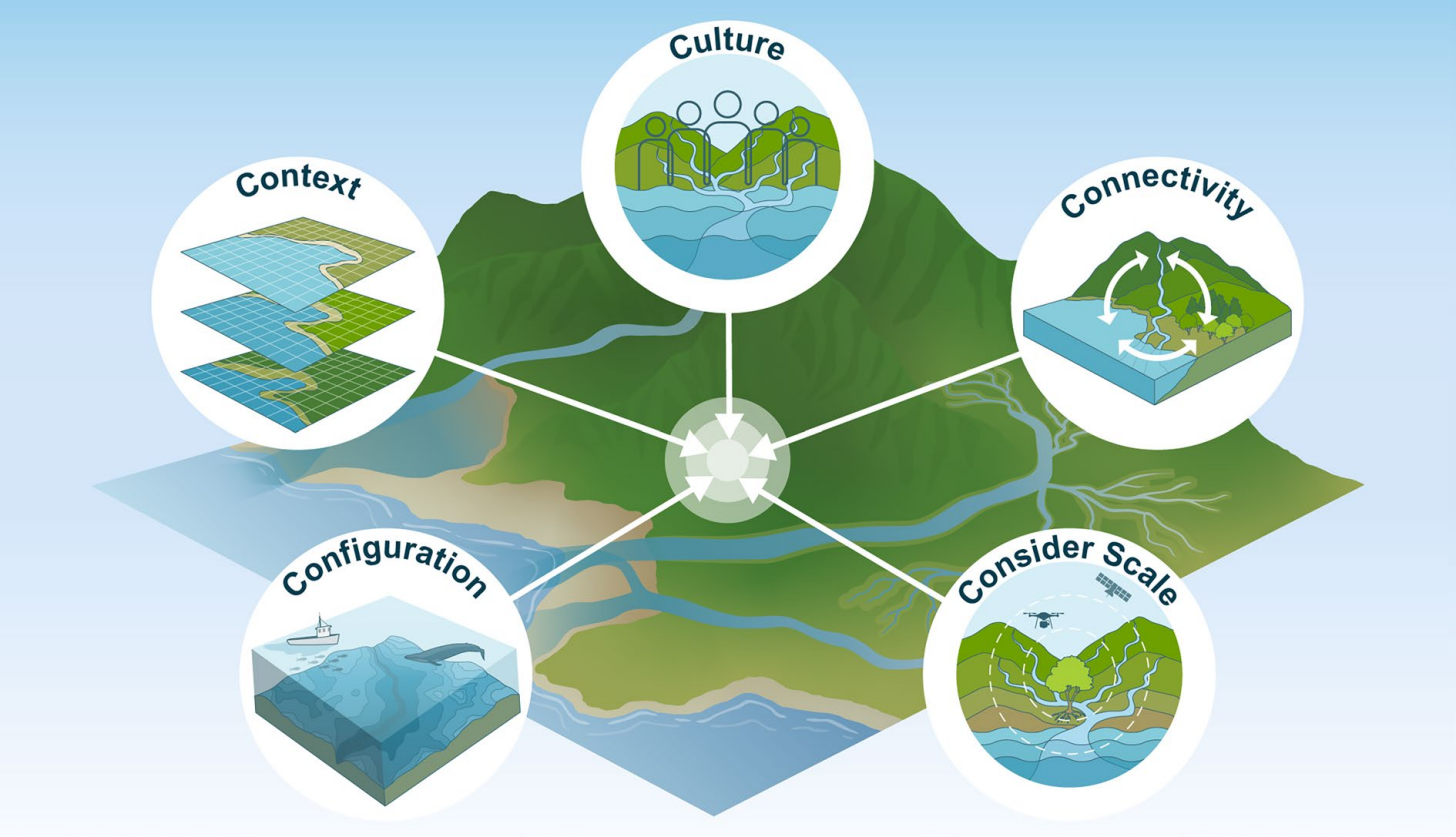
The 5Cs of seascape ecology—**C**ontext, **C**onfiguration, **C**onnectivity, **C**onsideration of scale, and **C**ulture—can help inform restoration ecology and enhance the design of other nature-based solutions

To outline research priorities for the field of seascape ecology, Pittman et al. (2021) distilled the science into four primary research themes: Context, Configuration, Connectivity, and Consideration of scale, known as the 4Cs. We tailor these key themes to marine restoration and add a 5th C for Culture. In doing so, we formally recognize the human connection to the sea and its importance by consciously deepening its integration into ecological research and restoration applications (Fig. 1; Table 1). Beyond the biophysical seascape, the ocean is also a lived space where complex, interconnected human–ocean relationships exist, creating unique senses of place (McNiven 2004; Ingersoll 2016; Said and Trouillet 2020; Pearson and Thompson 2023; Wedding et al. 2024). As seascape ecology matures, broadens in scope, and becomes more applied, both the theoretical and empirical aspects of the discipline can increasingly play a role in informing real-world decision-making. This solution-oriented shift is positioning seascape ecology as an operationally relevant science to support ecological restoration and regenerative development (Pittman et al. 2021). However, to ensure seascape ecology can help to achieve marine restoration goals, it is critical to establish a consistent language and framework for seascape restoration practitioners that complements existing restoration guidelines (e.g., the European Native Oyster Habitat Restoration Handbook and the Food and Agriculture Organization’s Standards of Practice to Guide Ecosystem Restoration) (Preston et al. 2020; Nelson et al. 2024). Here, we establish key terms for a common language and outline how the 5Cs of seascape ecology can directly inform marine restoration and enhance the design of these nature-based solutions to achieve cross-scale restoration targets (Fig. 1). Although defined separately, the 5Cs in nature are interdependent and operate synergistically across spatial, temporal, and ecological scales.

**Table 1:** Glossary of the key seascape restoration concepts and their definitions

Concept	Definition
Seascape ecology	The study of spatio-temporal patterns and ecological processes in heterogeneous coastal and marine environments, and how their interactions influence the social–ecological system and decision-making in restoration, conservation, and management practices.
Context	The geographic, spatio-temporal, ecological, cultural, and socio-economic conditions and events surrounding a focal site, including past, present, and future spatial patterns and dynamics.
Configuration	The spatial arrangement of physical structure including seafloor habitat mosaics, terrain topography, pelagic biological patchiness, and hydrodynamic features that influence ecological processes. Integrated within the spatial structure are elements of composition, which include the types of features and their variety and abundance in the seascape.
Connectivity	The way that the seascape facilitates or impedes natural processes such as movements of matter, energy, genes, and species between locations. Seascape connectivity is an emergent property resulting from complex interactions between the seascape’s biogeophysical structure and the attributes of the organisms, materials, or disturbances moving through it.
Consideration of scale	The understanding that no single spatial or temporal scale is characteristic of seascape ecology, whereby seascape patterns and processes operate across multiple scales in space and time, necessitating a multi-scale or cross-scale approach.
Cultural seascape	The diverse ways humans relate with seascape patterns and processes that influence beliefs, values, emotions, behaviors, stories, use, and sense of place.
Seascape restoration	A holistic approach to the restoration of multiple, interconnected habitats with the aim of recovering ecological connectivity, functionality and resilience. This approach should consider the dynamic interactions within a social-ecological system, including the related connectivity and interdependence among different species (including humans), habitats, and ecosystems.
Cross-habitat facilitation	Positive interactions, where processes in one habitat benefit another, involve physical, biological, and biogeochemical exchanges including effects like wave energy dampening, competition reduction, and nutrient cycling, extending to interactions across marine-terrestrial borders.

## Context

In seascape ecology, the terms 'context' and 'context dependence' refer to the conditions and events surrounding a focal site, encompassing knowledge of the past, present, and future spatial patterns and dynamics (Table 1). Research has uncovered high geographical variability that confounds many attempts to generalize ecological patterns and instead requires careful consideration of local and regional context (Bradley et al. 2020). For example, salt marshes are widely recognized as essential food sources and nursery habitats for fish, supporting community and food web stability. However, comparative studies of salt marsh habitats in different regional contexts reveal significant functional variability (Bradley et al. 2020). This variability is influenced by contextual factors such as tidal regimes, which regulate salt marsh availability (Rozas 1995) and, consequently, the capacity of these habitats to fulfil their presumed ecological functions. For instance, Ziegler et al. (2019) found that the proportions of salt marsh prey species in estuarine fish diets differed between regions, further underscoring the role of local environmental conditions in shaping habitat functions. Similarly, fish-mangrove linkages exhibit context dependency influenced by tidal regime and proximity to reefs and seagrasses (Pittman et al. 2007; Bradley et al. 2024).

It is likely that even two seemingly similar patches or seascapes can exhibit important differences in ecological form, function, and resilience due to geographic, temporal, ecological, cultural, and socio-economic variations in the surroundings. As such, spatial and temporal context, together with other seascape characteristics, is important in benchmarking restoration projects through appropriate reference site selection, the assessment of monitoring baselines, and the setting of realistic time-bound targets for outcomes (McNellie et al. 2020). The positive outcomes for regenerating species populations have been greater where landscape context has been considered in restoration site selection and design (Duncan et al. 2019; Rummell et al. 2024). Understanding the ecological context and shifts in species abundances and distributions through methods like habitat suitability modeling (e.g., Folmer et al. 2016; Stuart et al. 2021, 2024), and field surveys (e.g., Preston et al. 2020) provides critical information to support site-specific restoration efforts. For example, fieldwork by Preston et al. (2020) revealed that increases in the number of the invasive limpet *Crepidula fornicata* (Helmer et al. 2019) have negatively affected the recovery of the European native oyster *Ostrea edulis* in the United Kingdom's Solent seascape due to interspecific competition. Therefore, restoration methods were needed to create the enabling environmental context for the native oyster to complete its life cycle without inhibitory competition. Experiential learning and scientific reviews suggest that success in ecological restoration is highly context-dependent, with socio-economic and anthropogenic factors having multifaceted effects on restoration and conservation outcomes (Simenstad et al. 2006; Bayraktarov et al. 2020; Fraschetti et al. 2021).

Human influence has shaped the coastal seascape over millennia. The temporal context is crucial for understanding the historical, present, and future geographical distribution and condition of target ecosystems, habitats, or species. Restoration often aims to achieve a return to historical conditions, yet this approach is increasingly challenged by unprecedented changes and shifting baselines caused by anthropogenic drivers (Volpe et al. 2024). Therefore, a mindful and comprehensive temporal context is essential to create salient restoration practices and policies. For example, although currently in a state of collapse (zu Ermgassen et al. 2024), *O. edulis* historically dominated European coastlines in the last few centuries, forming extensive and structurally complex biogenic reefs (Thurstan et al. 2024). Understanding this historical perspective has been instrumental in fostering an evolving cultural acceptance that action is required to regenerate this highly valued complex habitat type. In addition, successful annual intertidal eelgrass (*Zostera marina*) restoration in the Dutch Wadden Sea (Govers et al. 2022; Gräfnings et al. 2023; 2024) has been achieved by considering both the spatial context (i.e., a highly suitable location that is propagule limited) and the temporal context (i.e., maintaining high genetic diversity through seed-based restoration to promote resilience).

A range of historical contexts and conditions can be considered for a restoration site. Even if ideal biophysical locations for restoration are chosen, context-dependent social pitfalls may arise and influence the outcomes. The social context and levels of public and political acceptance and support for restoration projects can fluctuate over different locations and periods, significantly influencing the success of these efforts (Miller and Hobbs 2007; Kenny et al. 2023). Achieving long-term restoration goals, therefore, necessitates that practitioners consider how their sites have been, and continue to be, influenced by socio-economic conditions, cultural values, and policy and governance structures (Stein et al. 2020). Horizon scanning must consider the potential for long-term positive outcomes by considering future threats from human activities and shifting ecosystem boundaries and habitat suitability levels under a warming global climate with a higher sea level (Reed et al. 2020; Lovelock et al. 2022). Understanding the social context for restoration in multi-functional coastal landscapes and seascapes requires a pluralistic and transdisciplinary approach (see Sect. 5. “Culture”). Explicit consideration of context and context dependence is imperative to maximize restoration efficiency and efficacy while ensuring sustained benefits for human and non-human recipients (Sheaves et al. 2021).

## Configuration

Seascape configuration refers to the spatial arrangement and orientation of habitat patches, including biogenic habitats, terrain topography, planktonic patchiness, and hydrodynamic features (e.g., fronts, currents, eddies, clines) that impact ecological processes (Table 1; Wedding et al. 2011; Kavanaugh et al. 2014; Borland et al. 2021). Recent advancements in remote sensing technologies and Geographic Information Systems (GIS) have simplified the quantification and visualization of seascape configuration (D’Urban Jackson et al. 2020; Lepczyk et al. 2021). Two-dimensional seascape structure can be mapped as a mosaic of discrete patches, like vector-based benthic habitat maps, whereas raster-based gradients are typically used to represent the three-dimensional heterogeneity of the marine environment, including bathymetry, rugosity, and temperature (Wedding et al. 2008; 2019; Diefenderfer et al. 2018). A suite of spatial pattern metrics, such as nearest neighbor distances, edge length, fragmentation, and seasonal percent cover, can be efficiently calculated from mapped data to provide a range of seascape configuration measures (Wedding et al. 2011; Borland et al. 2021; Davies et al. 2024). While these metrics originated in terrestrial landscape ecology and have been widely applied to land use management and conservation (Turner 1989; Gustafson 1998), their use in seascapes is complicated by three-dimensional dynamic marine conditions, including changing water volumes, wave action, and other environmental factors. To address these challenges, some metrics have been adapted for seascape ecology, while new ones have been developed (Wedding et al. 2011). Such novel seascape metrics can be incorporated into marine restoration plans and serve as valuable indicators for monitoring and evaluating the effectiveness of restoration efforts within the broader seascape context. For example, anticipating, designing, and monitoring edge effects is essential to understanding restoration function, avoiding negative edge effects, and optimizing positive ones (Humphreys et al. 2020). Patch edges and ecotones exhibit variable functions, often supporting diverse species assemblages and mediating predator–prey relationships and physical processes such as current speed and sediment accretion (Boström et al. 2011; Carroll et al. 2019).

Critically, restoration practitioners should keep in mind that the influence of seascape configuration may differ based on species, life stage, and the contextual setting. For instance, fishes associated with seagrasses, mangroves, and oyster and coral reefs exhibit individualistic, species, and life-stage specific responses to habitat patch abundance, proximity, clustering, and fragmentation (Nagelkerken et al. 2002; Pittman et al. 2004; Yeager et al. 2019; Grabowski et al. 2022; Santos et al. 2022). This variability has cascading effects on demographic structure, functional group representation, and rates of key ecological processes such as herbivory and predation (Overholtzer-McLeod 2006; Yeager et al. 2016; Swindells et al. 2017; Roff et al. 2019; Eggertsen et al. 2020). McAfee et al. (2022) describe efforts to restore lost oyster reefs (*Ostrea angasi*) along Adelaide’s highly modified coastline. Boulder reefs constructed to support *O. angasi* also provided substrata that facilitated the return of lost kelps (*Ecklonia radiata*). These kelps have a reciprocal relationship with oysters, contributing to shading, maintaining water chemistry, and suppressing understory algal species. Additionally, jute bags placed strategically on the leeward side of boulder reefs, where hydrodynamic energy is reduced, offered attachment substrata that aided the settlement of naturally recruiting seagrass seedlings (*Amphibolis antarctica*) (McAfee et al. 2022). If seascape restoration is properly planned with habitat configuration in mind, restoration practitioners can leverage ecological synergies to enhance restoration outcomes.

Terrestrial and coastal marine ecosystems are inextricably linked through their adjacent habitat configuration, which facilitates or impedes connectivity. As a result, land-based stressors can undermine the effectiveness of marine restoration (Akinsete et al. 2021). Therefore, effective land management and consideration of the land-sea habitat configuration is a crucial component of seascape restoration planning. To ensure effective coastal seascape restoration, programs must also evaluate how the physical configuration of adjacent terrestrial ecosystems and land uses influence the flow of organisms, sediments, nutrients, and pollutants, both directly and indirectly affecting restoration success (Ricart et al. 2015; Duncan et al. 2019; Saavedra-Hortua et al. 2020; Signa et al. 2020). Human use can be considered both harmful or helpful when documenting changes in the spatial configuration of restored habitats. For example, shoreline armoring, such as seawalls and breakwaters, alters wave dynamics by reflecting energy rather than absorbing it (Patrick et al. 2016). This erodes bottom sediments and sensitive benthic habitats, increasing nutrient transfer, and shoreline armoring can also disrupt water quality by suspending particulates, which harms both natural and restored submerged vegetation and local faunal communities. In contrast, the construction of stonewall fish traps or vywers (Goodwin 1946) along the South Coast of Cape Town, South Africa, from the last two centuries up to 5000BP (Kemp et al. 2009) support ecological communities with nearly double the species richness at the scale of the entire natural rocky shoreline (Kemp 2006). The existence, use, and restoration of vywers along the coastline have modified the seascape configuration, with measurable positive biodiversity outcomes and effects on species richness. Engaging local stakeholders to co-design restoration site placement that considers seascape configuration using local knowledge may benefit restoration project design (Suding et al. 2015).

## Connectivity

Connectivity, defined in landscape ecology as ‘the degree to which the landscape (or seascape) facilitates or impedes movement among resource patches’, is an emergent property arising from complex interactions between the seascape’s biophysical structure and the attributes of the organisms, materials, or disturbances moving through it (Table 1; Taylor et al. 2006). Ecological connectivity is typically divided into two key components: structural and functional connectivity. Structural connectivity focuses on the configuration of habitats and resource patches, without considering their influence on organism movement or ecological processes. Often measured as continuity among habitat patches, structural connectivity peaks when habitat patches share an edge and declines with increasing inter-patch distance. Functional connectivity considers how the context and the physical configuration of seascape features influence their ability (potential or actual) to support organism movement, gene flow, nutrient cycling, and other ecological processes (Tischendorf and Fahrig 2000; Taylor et al. 2006; Weins 2006). Thus, the functional connectivity of a given seascape varies among species, individuals, and developmental stages, depending on behavioral and physiological factors, life history traits, and the surrounding context, configuration, and quality of available patches (Pittman and McAlpine 2003; Ray 2005; Nagelkerken et al. 2015). Knowledge of seascape connectivity is not only key in the design of biodiversity positive restoration actions and prediction of the social and ecological consequences but also can be used to avoid and mitigate undesirable transmission of invasive species, disease, and other threats (Cantrell et al. 2020).

Structural connectivity can be readily quantified and modeled in GIS software based on mapped patterns. In contrast, functional connectivity is more challenging to directly measure (i.e., actual connectivity) or approximate using models (i.e., potential connectivity) (Calabrese and Fagan 2004). Depending on available resources and the focal organism's prevalence, body size, and spatio-temporal scales of movement, researchers have measured or inferred functional connectivity using tagging and tracking (Pittman et al. 2014; Appeldoorn and Bouwmeester 2022), graph theoretic or biophysical modeling (Treml and Halpin 2012; Rossi et al. 2014; Peterson et al. 2024; Stuart et al. 2024), and biogeochemical or genetic analyses of biological material (McMahon et al. 2013; Krueck et al. 2020). Such studies have contributed to a growing body of evidence demonstrating the central influence of connectivity on ecosystem functioning, genetic diversity, ecosystem sere provisioning, and resilience to disturbances (Mumby and Hastings 2008; Olds et al. 2016; Carr et al. 2017).

Crucially, maintaining ecological heterogeneity and biodiversity through the restoration of interconnected habitats—the 'portfolio effect'—will bolster resilience to local and global stressors (Schindler et al. 2015). Many examples now exist from coastal seascapes where positive synergistic interactions among seagrass, salt marsh, mangrove, coral reef, and oyster reef habitats have been attributed to ecological connectivity (Weinstein et al. 2005; Olds et al. 2018; McAfee et al. 2022; Preston et al. 2025). For example, Rummell et al. (2023) showed that wetland restoration success in eastern Australia, as measured by species richness, the abundance of fisheries target species, and the functional richness of fish and crustacean assemblages, was significantly improved by enhanced connectivity with the broader estuary. Over the 4-year monitoring period, wetland connectivity emerged as the most important predictor of co-benefit magnitudes, underscoring how connectivity, configuration, and context interact to shape restoration outcomes (Rummell et al. 2023).

Seascape ecology provides a suite of connectivity concepts, spatial tools, and indicators that aid in mapping, measuring, and monitoring structural and functional connectivity, as well as integrating these properties into restoration (Gillis et al. 2017; Pittman 2018). The flows and feedbacks within and between habitat patches are vital for maintaining and restoring diversity, function, and resilience (Preston et al. 2025). Therefore, to maximize restoration efficiency, it is essential to understand the multi-scale connectivity pathways that underpin the regenerative process. This enhanced understanding of connectivity should inform marine restoration design and implementation stages, focusing on rehabilitating and enhancing those ecological processes.

## Consideration of scale

Scale awareness is fundamental in seascape ecology research, yet this topic has been explored far less in the literature for coastal restoration than for terrestrial landscape restoration projects (Table 1). Growing societal recognition of the need to accelerate the scaling up of restoration efforts to restore coastal ecosystem integrity and connectivity has been further elevated by broad-scale changes due to global climate impacts. The scales at which we perceive and measure seascapes influence our understanding of their patterns and processes (Levin 1992; Kendall et al. 2011; Kavanaugh et al. 2014). Consequently, understanding scale and scale dependency is crucial for making informed restoration decisions (van Katwijk et al. 2016). Selecting the appropriate scale is essential for determining the extent of restoration and the resolution of ecosystem mapping and monitoring. When scaling up seascape restoration, the 5Cs will be essential to consider with respect to exploring costs, implementing transboundary cooperation and initiatives, and addressing other socio-cultural and -ecological system properties needed to integrate actions within broader landscapes and seascapes. Furthermore, when enacting this transdisciplinary cooperation at scale, including the human and ecological historical context is important.

Historically, animal ecology has advocated for scale selection based on meaningful scales of ecological processes, such as organism movements, a seascape unit of management interest, or a specific research question (Schneider 2001; Pittman and McAlpine 2003; Aston et al. 2019). Understanding the ecological consequences of coastal seascape structure, function, and dynamics that operate across multiple scales will help identify optimal spatial configurations for restoration design and support trade-off analysis in multi-functional seascapes. Further, restoration should be considered and envisioned at multiple levels—both on spatial and temporal scales—and implemented in a way that is mindful of human communities and their relationship with the sea. These initiatives can and should drive effective restoration at the local level first. These localities then act as essential interconnected nodes in a network of community practice, embedding restoration action across wider ecoregions and contributing to global restoration targets.

Therefore, we advocate for a multi-scale approach, acknowledging that no single spatial or temporal scale can be deemed characteristic for the application of seascape ecology (Wiens 1989; Holland and Yang 2016; Pittman et al. 2021). The multi-scale approach does not negate scale-dependent inquiries or methods but integrates them into a broader framework that accounts for cross-scale effects. This integration is crucial for multi-habitat restoration, facilitating cross-habitat interactions, and promoting positive ecosystem interactions. As such, we avoid using the terms *seascape scale* and *seascape level*, which are too often used as arbitrary and scale-ambiguous descriptors (Boström et al. 2011). Moreover, the temporal aspect of human activities, cosmologies, and the biophysical system are dynamic and complex at different scales (e.g., geological, decadal, inter-annual, seasonal, daily, hourly, and second-by-second).

**Fig. 2:**
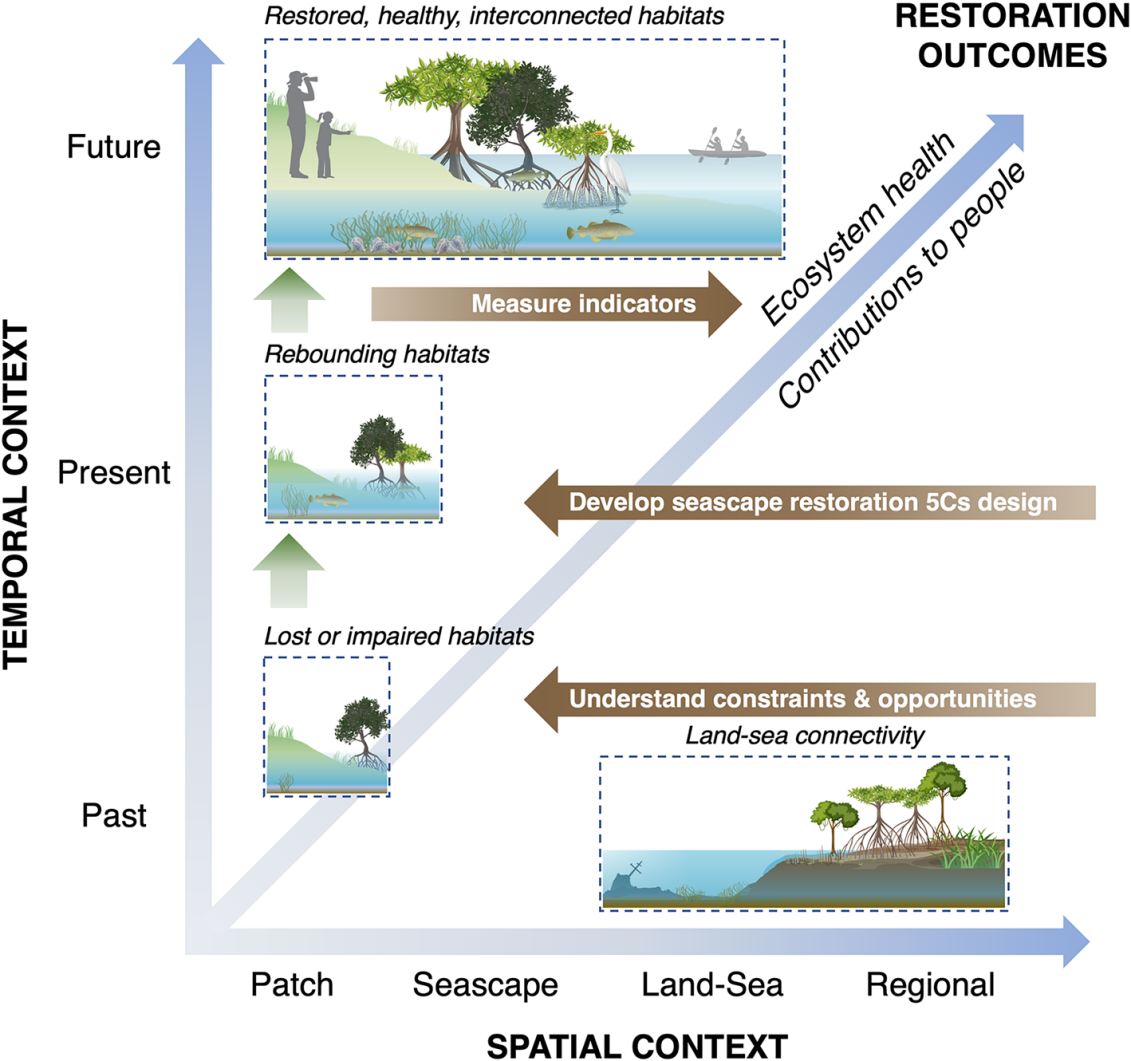
Diagram illustrating the integration of the 5Cs of seascape ecology (Context, Configuration, Connectivity, Consideration of scale, and Culture) to enhance strategies for scaling up coastal restoration across interconnected land–sea ecosystems. All five elements are key to consider across a range of spatial scales (X-axis) from habitat patches, seascape, land–sea interface, and at the regional-level. The restoration constraints and opportunities based on all five elements are important to consider when aiming to move across time (Y-axis) from the degraded habitats of the past to ensure seascape restoration best practices are applied to support rebounding habitats while key indicators are identified to monitor future restoration outcomes. This figure includes graphics from the Integration and Application Network (ian.umces.edu/media-library) and Vecteezy (https://www.vecteezy.com/)

Selecting the appropriate scale(s) for restoration remains challenging and depends on the system, methods, and target outcomes. For instance, field experiments have been conducted to determine the optimal seeding depth, injection density, and seed amount for intertidal seagrass restoration in the Dutch Wadden Sea (Gräfnings et al. 2023). Seagrass meadows exhibit both density and scale-dependent feedback (Maxwell et al. 2017), making scale a critical factor in restoration success (van Katwijk et al. 2016). Gilby et al. (2021) also demonstrated a multi-scaled systematic conservation planning framework for restoring multiple marine habitats in eastern Australia. Decision support tools like Marxan (Ball et al. 2009) and Zonation (Lehtomäki and Moilanen 2013) facilitate multi-scale assessments and restoration scenario testing before implementation. However, applications of these tools in restoration planning remain limited (Lester et al. 2020). It is prudent to define the ecological scales of influence within the surrounding seascape, identify the key ecological connections necessary for full recovery, and understand how the biophysical system interacts with local culture (Fig. 2).

## Culture

Cultural seascape ecology integrates the biophysical dimensions with the significance of place and focuses on how the reciprocal relationships between seascape patterns and processes link us as humans to the ocean (Puniwai 2015; Pittman et al. 2017; Wedding et al. 2024). Cultural seascape ecology is an emerging discipline exploring diverse human connections to the ocean, encompassing the meanings humans apply to the sea through their beliefs, values, emotions, behaviors, use patterns, stories, and associated systems (Wu 2010; Puniwai 2015; Wedding et al. 2024). Traditionally defined ocean boundaries, such as the tidal seawater limit or watershed boundaries, reflect biophysical concepts but may not align with human perceptions, cosmologies, and use patterns. We, therefore, expand the concept of cultural seascapes to encompass the regionally important interconnections with the ocean, which can extend inland through estuaries and farther still through a complex social–ecological network of processes particularly dense in coastal zones. By integrating and acknowledging culture—and disciplines that study culture, such as archaeology and anthropology—cultural seascape ecology aligns with and can draw from well-established frameworks. These frameworks illustrate how seafaring and coastal communities extend beyond their coastal footprint, both outward to sea and inland, encompassing economies, resource uses, and spiritual practices (Westerdahl 1992, 2012).

The cultural seascape ecology perspective underscores how humans affect and are affected by seascapes, with these relationships shaping coastal management and restoration approaches. The relevance and potential benefits of cultural seascape ecology for coastal restoration are only just emerging. Perceiving cultural seascapes as adaptive social–ecological systems sets the foundation for understanding how human values for ocean places evolve amid environmental and cultural changes—insights that could enhance restoration design (Fleming et al. 2019). In balanced social–ecological systems, biophysical restoration can be interwoven with biocultural restoration and resilience by acknowledging, restoring, and safeguarding the cultural relationships to place (Table 1; Morishige et al. 2018). Including consideration of culture can shift the aims of sustaining social–ecological systems to enabling the cultivation of thriving living systems that enhance health, well-being, and happiness (Gibbons et al. 2018).

**Fig. 3:**
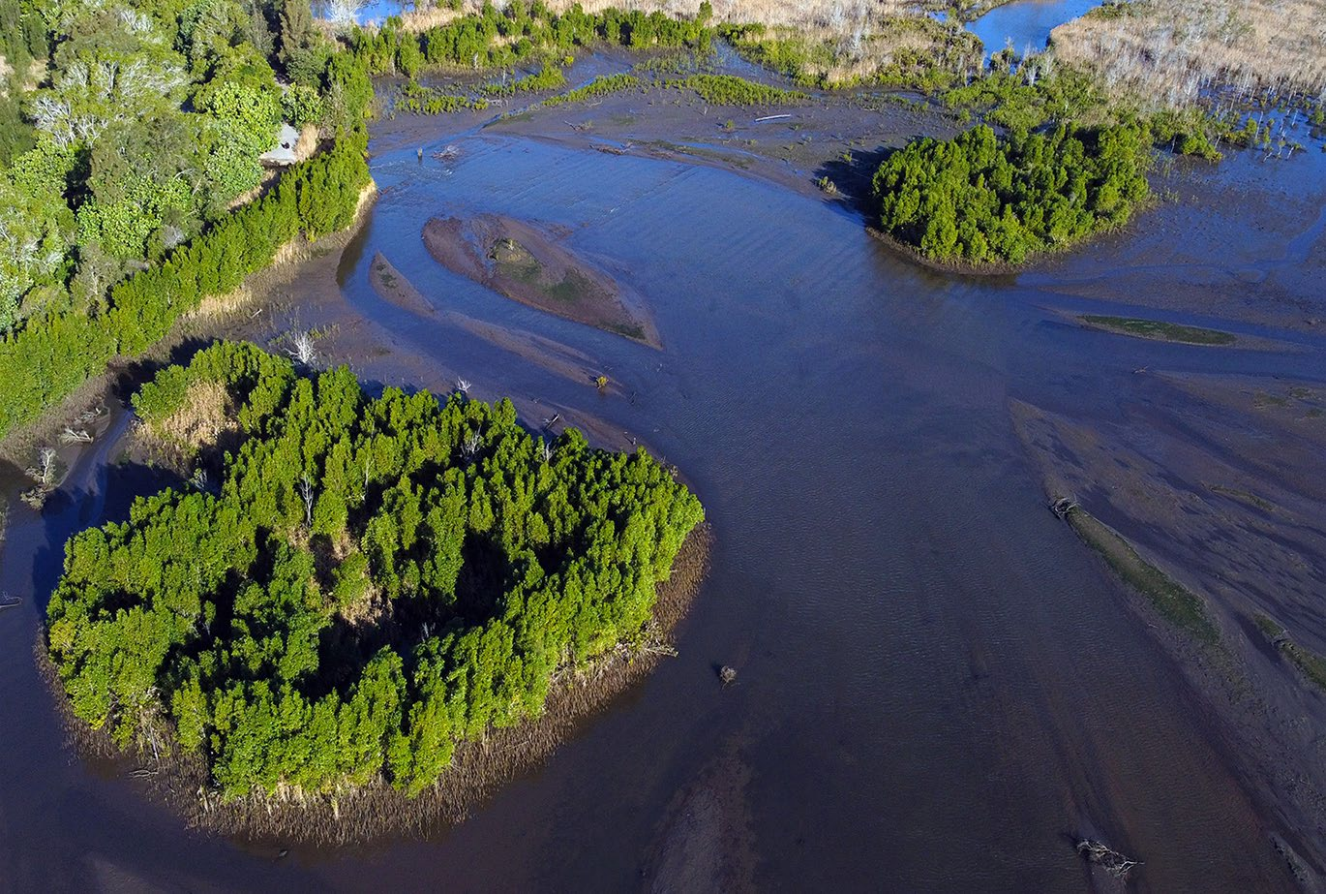
Recovering vegetation patches in the Blue Heart Sunshine Coast, eastern Australia, differ in their context, configuration, and connectivity. These seascape attributes affect the recovery of biodiversity, fisheries targets, and ecosystem functioning

Inclusive and pluralistic contributions of knowledge that consider diverse perspectives and stakeholders can enhance restoration performance beyond habitat creation (Abelson et al. 2020). Partnering with Traditional Owners offers a pathway toward more socially and culturally just restoration (McNiven and Russell 2005). For instance, the Darumbal people of Australia’s Shoalwater Bay—with institutional knowledge spanning over 9000 years—link Dreaming stories to sea ownership and coastal change (McNiven 2004; McNiven and Russell 2005). Effective seascape recovery requires both ecological expertise and an understanding of present and past cultural contexts, making the involvement of diverse partners essential. However, few case studies demonstrate effective restoration co-design practices, underscoring the need for greater funding to support such efforts to enhance co-development and knowledge sharing in restoration projects (Fig. 3).

Coastal communities often hold detailed knowledge of seascape dynamics, which is vital for selecting restoration targets, methods, and metrics (Opdam 2020). Beyond local ecological knowledge, material culture (e.g., shipwrecks, harbor structures, and shell middens) is a tangible way for restoration practitioners to incorporate human culture into biophysical restoration. For instance, ancient burial mounds have been used to reintroduce grassland plant species as monuments that celebrate cultural heritage and biodiversity restoration for the public (Valkó et al. 2018). Restoration efforts integrating material culture, history, and local knowledge can bridge jurisdictional divides, » environmental recovery (Weinstein 2008; Kenny et al. 2023).

Box 1: Case study: Blue Heart Sunshine Coast, a restoring tidal wetland seascape in eastern Australia The Blue Heart Sunshine Coast is a diverse seascape restoration site in the tidal floodplain of the Maroochy River catchment and estuary in Queensland, eastern Australia, covering an area of 5000 ha (Sunshine Coast Regional Council 2024). It encompasses a range of restoration sites, conservation areas, natural and rural lands. The Kabi Kabi people, Traditional Owners of the land, have maintained deep connections with the Maroochy River floodplains for millennia—shell middens in the area have been dated to 9900 BP—making it a region of significant cultural heritage (Wilson and Pearce 2017). Restoration has commenced at multiple locations in the region and is expanding rapidly as local and state governments work in partnership with utility companies, landowners, and the community to restore former farmlands to tidal wetlands and deliver diverse environmental, social, and economic benefits. Several alternative land uses have been implemented through levy-funded land acquisition by environmental projects to support community empowerment and engagement. Recreation trails have been developed for locals and sustainable tourism, alongside a solar farm and a blue carbon pilot project established on council-owned land. The Sunshine Coast Council actively engages landholders, residents, and local organizations through information sessions to ensure community involvement in restoration planning, with particular interest from the community in blue carbon farming as an alternative to traditional agriculture (Sunshine Coast Regional Council 2024). Such co-design of restoration projects and alignment of objectives with local preferences has been shown to enhance social and environmental outcomes (Löfqvist et al. 2023). Restoration sites are selected based on their availability (i.e., land ownership), and their suitability for target habitats and ecosystems (i.e., inter-connected mangroves, tidal marsh, and supra-tidal swamp forest) using data derived from digital elevation and hydrodynamic models. The oldest restoration site in the region, a 190-ha wetland seascape, has been recovering for approximately 6 years and has experienced increases in the extent of mangroves (2 ha), tidal marsh (5 ha), and supra-tidal swamp forest (9 ha). Changes in this wetland seascape have also led to the rapid recovery of aquatic fauna, which has scaled with the extent of tidal inundation and wetland habitat availability. Spatial patterns in the distribution of diversity, fisheries target species, and ecosystem functioning are positively linked to the context and configuration of recovering mangrove and tidal marsh patches and the level of connectivity with other wetlands (Rummell et al. 2023; 2024).

Nearby archaeological and anthropological work conducted with the Darumbal people and the Torres Strait Islanders may offer a valuable framework for establishing partnerships on the Sunshine Coast. These partnerships are essential for fostering inclusivity (McNiven and Russell 2005). Restoration efforts in these areas, such as outplanting seagrass seedlings in Shoalwater (Verduin et al. 2013) or wetland assessment and management in the Torres Straits (Waltham et al. 2018), benefitted from collaboration with local groups. For instance, Waltham et al. (2018) found that recording traditional language names for surveyed species helped communicate scientific findings while also enhancing the understanding of local food sources. Additionally, the fencing off of feral deer from local wells—while allowing access for the community to a culturally important wetland site—resulted in the water supply lasting months longer and into the dry season. These examples underscore the importance of communication and partnership in seascape restoration.

## Discussion

The effects of context, configuration, connectivity, and scale are pervasive across coastal seascapes and have been reported for a diversity of species, assemblages, and functions in tropical, subtropical, and temperate waters. These features of seascapes influence the design and performance of marine conservation areas, but are not yet well integrated into restoration design. There is a growing need to broaden restoration aims from habitats and ecosystems to entire seascapes and their related cultural components. However, supporting evidence of the effects of context, configuration, connectivity, consideration of scale, and culture on the restoration of ecological functioning is needed and remains scarce. Establishing the scientific rationale for seascape restoration remains an important foundational step. We encourage restoration practitioners and funders to shift from viewing habitats in isolation, or as simple interlinked pairs of ecosystems, and instead move towards prioritizing and restoring interconnected seascape mosaics as part of a wider social-ecological system. We recognize that practical limitations exist, as many coastal seascapes have limited mapping products and cultural data to provide the necessary foundational information. Restoration practitioners must either rely on the best available data or generate baseline products using high-resolution, low-cost modeling or remote sensing techniques (see GBF Target 21, CBD 2022; Rummell et al. 2022). As data availability and resolution improve, this holistic seascape approach to restoration will be pivotal for supporting the recovery of diverse biological communities, enhancing key functions and ecosystem services, and optimizing the flow of ecological benefits.

A fourth dimension of the marine environment—time—should also be considered, prompted by the influence of a changing climate that is driving oceanic temporal variability (Doxa et al. 2022), with subsequent impacts on seascape configuration, patch quality, and connectivity between seascape features. This climate-driven fourth dimension highlights the need to consider temporal variability in seascape restoration, something now included in emerging approaches such as dynamic ocean management (Maxwell et al. 2015) and climate-smart marine spatial planning (Frazão Santos et al. 2024). Dynamic ocean management is an approach that can adapt spatially and temporally to changes in the ocean and its users in response to near-real-time biological, oceanographic, social, and economic data (Maxwell et al. 2015). For example, species habitat ranges and connectivity corridors shift in response to climate-induced warming and changes in oceanic circulation (Wilson et al. 2016). Dynamic ocean management approaches share commonalities with seascape restoration ecology through the key biological and socio-economic considerations that together make a ‘seascape.’ Moving forward, incorporating a dynamic component into the 5Cs could create a more adaptive framework for seascape restoration programs that could be situated in a broader MSP and more resilient to climate-driven change.

The 5Cs framework can be employed throughout a seascape restoration project, including during the stages of planning and design, determining methods and metrics, monitoring, reporting, and making iterative improvements. We recommend that seascape restoration metrics or indicators used are spatially relevant to increase the practical viability where the 5Cs can be embedded in coastal restoration. Tools to map, model, and monitor functional connectivity—such as tagging and tracking, habitat suitability models, and environmental DNA—offer robust information in support of the protection and management of the seascape and its resources (Balbar and Metaxas 2019; Stuart et al. 2021; 2024). These techniques should be used alongside restoration guidelines to ensure complementary efforts and achieve additionality. To overcome one of the biggest challenges to the application of social–ecological knowledge, restoration projects must build a better understanding of operational processes and decision-making in restoration practice.

A transdisciplinary approach to restoration planning will be key. Cultural seascape ecology can support the needed community-driven visions for healthier coastal environments through reciprocal and regenerative approaches that nurture human–ocean relationships and ecosystem recovery (Wedding et al. 2024). Better integration of humanistic perspectives from the social sciences into an adaptive social–ecological systems framework will help to achieve the triple-win outcomes desired by nature-based solutions such as restoring climate regulation, biodiversity gain, and co-benefits for humans. Especially important for impacted urban coastal areas, closer collaboration between seascape restoration practitioners and local communities could improve inclusive and adaptive regenerative design that ensures meaningful functional integrity and future resilience. We suggest this engagement process integrates public involvement and local knowledge, as well as an understanding of the local social–political context—inclusive of a longer temporal scale—to build an appropriate solution-oriented approach (Opdam et al. 2018).

Understanding coastal system dynamics and connections across the land–sea interface can improve the design of restoration interventions. With the global expansion of science-informed restoration best practices, opportunities exist for restoration projects to partner with scientists (academic and citizen) to conduct broad-scale experiments. Restoration projects provide important opportunities for broad-scale manipulative experiments linking patterns and processes, which have played an important role in terrestrial landscape ecology but are still rare in seascape restoration projects (Gellie et al. 2018; Wiersma 2022). Future work could benefit from seascape restoration projects that provide living labs (Lupp et al. 2020; Franke et al. 2023) to monitor and evaluate seascape restoration science and practice.

## Conclusion

When moving towards implementing seascape restoration in practice, we recommend four key considerations. First, the focus of restoration needs to broaden from individual target habitats or pairs of linked adjacent habitats, to the recovery of interconnected ecosystems in diverse, functioning seascapes. Despite mounting evidence for holistic and multi-habitat approaches to seascape restoration and management (Oleson et al. 2018; McAfee et al. 2022; Vozzo et al. 2023), single-habitat and paired-habitat restoration methods remain dominant (Gilby et al. 2018). Moving forward, efforts must cover the entire spectrum of seascape habitats, especially research on connectivity, which is currently skewed toward charismatic species and visible biogenic habitats like coral reefs and coastal wetlands. This conceptual and operational shift is particularly important for the recovery of species that benefit from access to resources across multiple patch types during daily, seasonal, or reproductive migrations. Second, this means considering each of the 5Cs from the beginning of the restoration process. This is essential to inform the selection of appropriate and interlinked restoration targets, goals, and objectives. At this point, integrating cultural perspectives on seascapes, restoration, and ecology will become crucial. The effects and potential benefits of restoration should be assessed from the start with a monitoring design informed by the 5Cs. This design must quantify the impact of seascape features on restoration outcomes and be conducted at an appropriate frequency to ensure alignment with project goals and objectives over time. Funders must recognize that the time frame and resources required to study broad-scale restoration projects often exceed the typical funding duration. Third, effective seascape restoration planning requires maps and habitat suitability models for target ecosystems. These should be generated at a resolution and extent that aligns with project goals and objectives, and incorporate scenarios that explore the effects of different seascape contexts, configurations, and connectivity on restoration outcomes. Finally, transdisciplinary collaborations between scientists and practitioners of restoration projects will be essential to ensure the co-development of restoration goals.

The restoration-focused goals of the Kunming-Montreal Global Biodiversity Framework have been informed by landscape ecology. We perceive a growing role for landscape ecology concepts and methods in evidence-based coastal restoration ecology in alignment with the International Association for Landscape Ecology (IALE) core mission “to transcend boundaries and disciplines, to collaboratively build theory and develop knowledge of landscape pattern and process, to develop integrative tools and apply them to real landscape situations, and to apply our science towards solving problems”. However, challenges remain for the science to adequately support the successful practice of coastal seascape restoration. The recent adoption of the EU Nature Restoration Law represents a significant opportunity and call to action to scale up marine restoration. The 5Cs of seascape ecology—Context, Configuration, Connectivity, Consideration of scale, and Culture—can enhance the design of nature-based solutions to achieve global restoration targets and support local coastal ecosystem recovery. As seascape ecology matures, broadens in scope, and becomes more applied, both the theoretical and empirical aspects of the discipline can increasingly play a role in informing real-world decision-making. Integrating landscape and seascape ecology concepts and methods into coastal restoration will enable the effective scaling up of regenerative actions that is urgently required for a thriving and resilient future.

## Data Availability

No datasets were generated or analysed during the current study.
